# The dosimetric impact of replacing the TG-43 algorithm by model based dose calculation for liver brachytherapy

**DOI:** 10.1186/s13014-020-01492-9

**Published:** 2020-03-09

**Authors:** Anna Sophie Duque, Stefanie Corradini, Florian Kamp, Max Seidensticker, Florian Streitparth, Christopher Kurz, Franziska Walter, Katia Parodi, Frank Verhaegen, Jens Ricke, Claus Belka, Gabriel Paiva Fonseca, Guillaume Landry

**Affiliations:** 1 Department of Radiation Oncology, University Hospital, LMU Munich, Marchioninistraße 15, Munich, 81377 Germany; 2 Department of Medical Physics, Faculty of Physics, Ludwig-Maximilians-Universität München, Am Coulombwall 1, Garching, 85748 Germany; 3 Klinik und Poliklinik für Radiologie, Klinikum der Universität München, Marchioninistraße 15, Munich, 81377 Germany; 4 Department of Radiation Oncology (MAASTRO clinic), GROW – School for Oncology and Developmental Biology, Maastricht University Medical Centre, Dr. Tanslaan 12, Maastricht, 6229 ET The Netherlands; 5 German Cancer Consortium (DKTK), Munich, Germany

**Keywords:** Radiotherapy, Interstitial liver brachytherapy, HDR Ir-192 brachytherapy, Model-based dose calculation algorithm, Monte Carlo simulation, TG43, TG186

## Abstract

**Purpose:**

To compare treatment plans for interstitial high dose rate (HDR) liver brachytherapy with ^192^Ir calculated according to current-standard TG-43U1 protocol with model-based dose calculation following TG-186 protocol.

**Methods:**

We retrospectively evaluated dose volume histogram (DVH) parameters for liver, organs at risk (OARs) and clinical target volumes (CTVs) of 20 patient cases diagnosed with hepatocellular carcinoma (HCC) or metastatic colorectal cancer (mCRC). Dose calculations on a homogeneous water geometry (TG-43U1 surrogate) and on a computed tomography (CT) based geometry (TG-186) were performed using Monte Carlo (MC) simulations. The CTs were segmented based on a combination of assigning TG-186 recommended tissues to fixed Hounsfield Unit (HU) ranges and using organ contours delineated by physicians. For the liver, *V*_5Gy_ and *V*_10Gy_ were analysed, and for OARs the dose to 1 cubic centimeter (*D*_1cc_). Target coverage was assessed by calculating *V*_150_, *V*_100_, *V*_95_ and *V*_90_ as well as *D*_95_ and *D*_90_. For every DVH parameter, median, minimum and maximum values of the deviations of TG-186 from TG-43U1 were analysed.

**Results:**

TG-186-calculated dose was found to be on average lower than dose calculated with TG-43U1. The deviation of highest magnitude for liver parameters was -6.2% of the total liver volume. For OARs, the deviations were all smaller than or equal to -0.5 Gy. Target coverage deviations were as high as -1.5% of the total CTV volume and -3.5% of the prescribed dose.

**Conclusions:**

In this study we found that TG-43U1 overestimates dose to liver tissue compared to TG-186. This finding may be of clinical importance for cases where dose to the whole liver is the limiting factor.

## Background

Since the publication of the report of the American Association of Physicists in Medicine’s (AAPM) Task Group (TG) 186 [1], which advocates the use of model-based dose calculation (MBDC) as replacement to the water kernel superposition algorithm recommended by the AAPM’s TG-43U1 [2], there has been a concerted effort to identify brachytherapy treatment sites necessitating more sophisticated dose calculations. Treatment sites analysed so far include prostate [3], breast [4–7], head and neck [8], esophagus [6] and gynecologic regions [6, 9, 10]. To our knowledge there are no studies on the effect of MBDC for liver brachytherapy. MBDC make use of tissue composition and mass density as estimated from x-ray computed tomography (CT) images of the patient to account for differential attenuation, scattering and absorption between tissues. An important application of MBDC is also applicator modelling [1]. Algorithms for MBDC of current interest for ^192^Ir brachytherapy include collapsed-cone point kernel superposition [11–13], grid-based Boltzmann solvers [14, 15] as well as reference Monte Carlo (MC) simulation [16]. Different treatment planning systems (TPS) have adopted some of the algorithms listed above [13, 17]. In the context of ^192^Ir high dose rate (HDR) brachytherapy, the use of MBDC is particularly important for treatment sites in the vicinity of tissue/air or tissue/lung interfaces, and wherever variations of tissue density play an important role [1].

The treatment of liver malignancies by CT-guided HDR brachytherapy, where fluoroscopy-CT is employed for catheter insertion and three dimensional (3D) breath-hold CT for treatment planning, is associated with encouraging clinical results for liver metastases as well as hepatocellular carcinoma [18, 19]. It serves as an alternative to traditional treatment methodologies such as stereotactic body radiation therapy (SBRT) [20–24], transarterial chemoembolization (TACE) [25, 26] and radiofrequency ablation (RFA) [27] for nonresectable tumours. At the hospital of the Ludwig-Maximilians-Universität München (LMU Munich), 196 patients were treated with interstitial brachytherapy for liver malignancies in 2018, and coincidentally also in 2019.

The goal of this study was to assess whether MBDC are required for accurate dosimetry of liver brachytherapy given the organ’s proximity to the lung, its higher density than water, and the proximity of organs at risks (OARs) such as the stomach or duodenum (in addition to healthy liver tissue). MBDC were retrospectively compared to TG-43U1 calculations for liver cancer cases treated with ^192^Ir HDR brachytherapy, by employing CT-based, gold-standard MC simulations performed with the AMIGOBrachy [28] platform. Dose volume histogram (DVH) indices for the clinical target volume (CTV), for the OARs and for the whole liver were compared between TG-186 and TG-43U1 dose distributions.

## Materials and methods

### Clinical cases

This study retrospectively evaluated 20 treatment plans of 18 patients (15 males and 3 females) who were diagnosed with either metastasized colorectal cancer (mCRC) or hepatocellular carcinoma (HCC) and were treated with interstitial CT-guided brachytherapy at the university hospital of the LMU Munich. The patients had one (10 cases) or multiple (10 cases) lesions in the liver. The total number of treated lesions was 41. CTVs showed a high variation in volume, from less than 1 c*m*^3^ up to about 280 c*m*^3^, and as a consequence the number of dwell positions per CTV ranged from less than 10 to 100. Doses of 12, 15, 20 or 25 Gy were prescribed and administered in a single fraction. The cases were selected to ensure a high variety and even distribution of prescription doses, number of CTVs, OAR types and medical diagnosis. Furthermore, no unused catheters were present and at least one OAR was delineated for every case. Table 1 contains more detailed information about the patients’ characteristics.

**Table 1:** Clinical situation of the 18 patients analysed in this study

Case index	Patient	Gender	Treated lesions	Diagnosis	pr. dose (Gy)	OARs
i	1	m	3	mCRC	25	esophagus, stomach
ii	2	m	4	HCC	15	bowel, esophagus, heart, kidney, stomach
iii	3	f	3	mCRC	25	bowel, kidney, stomach
iv	4	m	3	mCRC	25	bile duct, bowel, colon, stomach
v	4	m	1	mCRC	20	colon, duodenum, kidney, stomach
vi	5	m	2	HCC	12	colon, stomach
vii	6	m	1	HCC	15	heart, stomach
viii	7	m	1	HCC	12	bile duct, bowel, gall bladder, kidney
ix	8	m	1	mCRC	25	bile duct, colon, duodenum
x	8	m	1	mCRC	25	heart, oesophagus, stomach
xi	9	m	1	HCC	15	heart
xii	10	m	1	HCC	12	bowel, stomach
xiii	11	m	3	mCRC	20	bowel, duodenum, stomach
xiv	12	m	1	mCRC	25	colon, kidney
xv	13	m	1	mCRC	20	bowel, duodenum, stomach
xvi	14	m	5	mCRC	25	colon, duodenum, heart, stomach
xvii	15	f	3	mCRC	20	colon, stomach
xviii	16	m	3	HCC	15	heart
xix	17	m	2	mCRC	25	esophagus, heart, stomach
xx	18	f	1	mCRC	25	heart

Patients 4 and 8 were treated twice with different treatment plans, so the total number of analysed cases was 20. Patients were diagnosed with either metastatic colorectal cancer (mCRC) or hepatocellular carcinoma (HCC). Prescription (pr.) doses ranged from 12 to 25 Gy. The last column lists the OARs which were considered for each case

Prior to treatment, brachytherapy catheters were inserted under fluoroscopy-CT guidance and local anaesthesia using a Seldinger technique. To achieve accurate placement of a catheter, a hollow gauge needle was first placed inside the lesion, then a stiff angiography guide wire was inserted into the needle. An angiography sheath was then inserted over the guide wire and the brachytherapy catheter was thereafter inserted into the angiography sheath [18, 29].

After successful placement of the catheters, a contrast-enhanced planning CT (SOMATOM Definition Edge, Siemens) was acquired using an end-of-exhale breathhold technique. Catheter reconstruction, treatment planning and dose optimization were performed with the Oncentra ^*Ⓡ*^ Brachy treatment planning system (Elekta AB, Stockholm, Sweden) which uses the dose calculation algorithm of the TG-43U1 protocol. For each case the CTVs were delineated, which correspond to the GTVs extended by a 0-1 mm margin. As there are few setup or motion uncertainties in brachytherapy, the planning target volume (PTV) is equal to the CTV. Furthermore, the whole liver and a set of OARs were contoured for clinical treatment planning, from which the most relevant were selected for analysis in this study. The stomach was the most frequent OAR (14 cases), followed by heart (8 cases), bowel and colon (7 cases), right kidney and duodenum (5 cases), esophagus (4 cases), bile duct (3 cases), and gall bladder (1 case). Table 1 lists the relevant OARs for each case.

Brachytherapy treatment was administered with an HDR afterloading system (Flexitron, Elekta AB, Stockholm, Sweden) using an ^192^Ir HDR source.

### MC simulations

MC simulations were performed with MCNP6 (Monte Carlo n-Particle, version 1.0 [30]), which is integrated in the auxiliary software AMIGOBrachy [28]. A phase space source was used for calculation, validated in a previous study [31]. As a TG43-U1 surrogate, we employed an MC simulation with the whole patient geometry set to water (*D*_w,w_). We used this instead of the TG-43U1 dose from the clinical TPS to avoid introducing biases which could be caused by TPS-specific aspects such as TG43-U1 parameter interpolation or cable modelling. For assessing dose transported in medium and scored in water (*D*_w,m_) or scored in the medium itself (*D*_m,m_), MC simulations were performed on a heterogeneous patient model (TG-186) based on the CT geometry. The CT images were segmented using auxiliary software, AMIGOBrachy [28], to create voxel geometries consisting of 5 tissues (lung (*ρ* = 0.26 g/cm^3^), mean adipose tissue (*ρ* = 0.95 g/cm^3^), mean male soft tissue (*ρ* = 1.03 g/cm^3^), mean female soft tissue (*ρ* = 1.02 g/cm^3^), liver (*ρ* = 1.06 g/cm^3^), and cortical bone (*ρ* = 1.92 g/cm^3^)), with compositions and densities recommended by TG-186 [1], and air (*ρ* = 0.0012 g/cm^3^).

The segmentation was performed by assigning homogeneous densities corresponding to the tissues specified above to fixed Hounsfield Unit (HU) ranges (see Table 2). We chose to assign reference densities instead of densities derived from a CT calibration curve to account for variations in density in organs which contained contrast agent, since during the time period from CT acquisition to treatment administration the contrast agent concentration decreased in the patient. In every case the liver was overwritten with a homogeneous density and composition according to TG-186, using the delineated organ contours. Parts of certain OARs (heart, kidney) were initially misassigned due to a high concentration of contrast agent. In these cases, the affected voxels were overwritten with mean male/female soft tissue density and composition. 

MC calculations were performed on an interpolated version of the CT grid. Voxel dimensions within transversal planes of the CT grid ranged from 0.61 mm to 0.98 mm for the different cases and were deemed small enough for direct adoption to the dose calculation grid of the simulation. CT slice resolution ranged from 2 mm to 3 mm and was interpolated to enable a dose grid slice resolution of 1 mm. Dose values were approximated as collision kerma assuming charged particle equilibrium, so secondary electrons were not transported [32, 33]. Simulations were performed using a track length tally and adopting mass-energy absorption coefficients (*μ*_*en*_/ρ) from the National Institute of Standards and Technology (NIST) [34] for either water (*D*_w,m_) or medium (*D*_m,m_) to convert photon energy fluence to collision kerma/dose. Calculations were performed using the ^192^Ir photon spectrum available from the National Nuclear Data Center [35]. The calculation method has been described in detail by Fonseca *et al* [28,31] and was also used by several authors [36–38]. The dose was cut off at a maximum value of 200 Gy for better comparison with the TPS, which uses this cutoff as a standard. MC uncertainties for all cases were assessed by calculating the voxel-wise standard deviation of repeated MC simulations and determining its maximum value within the 30%-isodose line (relative to prescription dose) for *D*_w,w_ and *D*_w,m_. *D*_m,m_ uncertainties are of the same magnitude as *D*_w,m_ uncertainties for the calculation method used in this study. We set the number of particles (up to 4x10^10^ for the different cases) such that *D*_w,w_ standard deviations within the 30%-isodose line were all below 0.8% and *D*_w,m_ standard deviations below 1.3%. To analyse the influence of these uncertainties on DVH parameter estimation we repeated the simulation for case iv (chosen arbitrarily) after increasing the number of histories by a factor of 10 and compared the DVH parameters with the results for the original number of histories. This led to deviations in the DVH-parameters that were all smaller than 0.2% of the respective DVH parameter value and were therefore considered negligible.

**Table 2:** Hounsfield Unit thresholds used for tissue segmentation

HU range	Assigned tissue
-1024 to -900	Air
-899 to -200	Lung
-199 to 0	Mean adipose tissue
0 to 200	Mean male/female soft tissue
201 to 2000	Cortical bone

### Dosimetric comparison

To evaluate differences between TG-43U1 and TG-186 in terms of clinically relevant quantities, DVH parameters were calculated for the liver, OARs and CTVs. To assess liver toxicity, the fractional liver volumes receiving at least 5 Gy or 10 Gy were calculated (*V*_5Gy_ and *V*_10Gy_, respectively). For each OAR, the dose to 1 cubic centimeter (*D*_1cc_) was calculated. For the CTVs we considered the fractional volumes receiving at least 150%, 100%, 95% or 90% of the prescribed dose (*V*_150_, *V*_100_, *V*_95_ and *V*_90_, respectively), and the doses that were administered to 95% or 90% of the CTV volumes (*D*_95_ and *D*_90_, respectively).

We determined for each DVH parameter the median, minimum and maximum values over all treatment plans, calculated with *D*_w,w_. To analyse the deviations between TG43-U1 and TG-186 we considered the absolute difference of TG-186-calculated parameters from *D*_w,w_. Median, minimum and maximum of these deviations were calculated. For liver and OARs, the DVH parameters were compared to clinical tolerance limits.

## Results

We report the comparison of *D*_w,w_ and *D*_w,m_ in the following subsections, divided into liver, OAR and CTV DVH parameter analysis. All of the reported results were obtained by MC simulations. Tables with the values for *D*_m,m_ were not included, since the results were similar to the comparison of *D*_w,m_ and *D*_w,w_ and thus do not provide additional insights. For completeness they can be found in the Appendix (Tables 6, 7 and 8).

For an easier comparison between parameters, we also included the deviations from TG-43U1 in percentage of the corresponding parameter value. The tables can be found in the Supplementary material (S1, S2 and S3).

### Liver dose

**Fig. 1:**
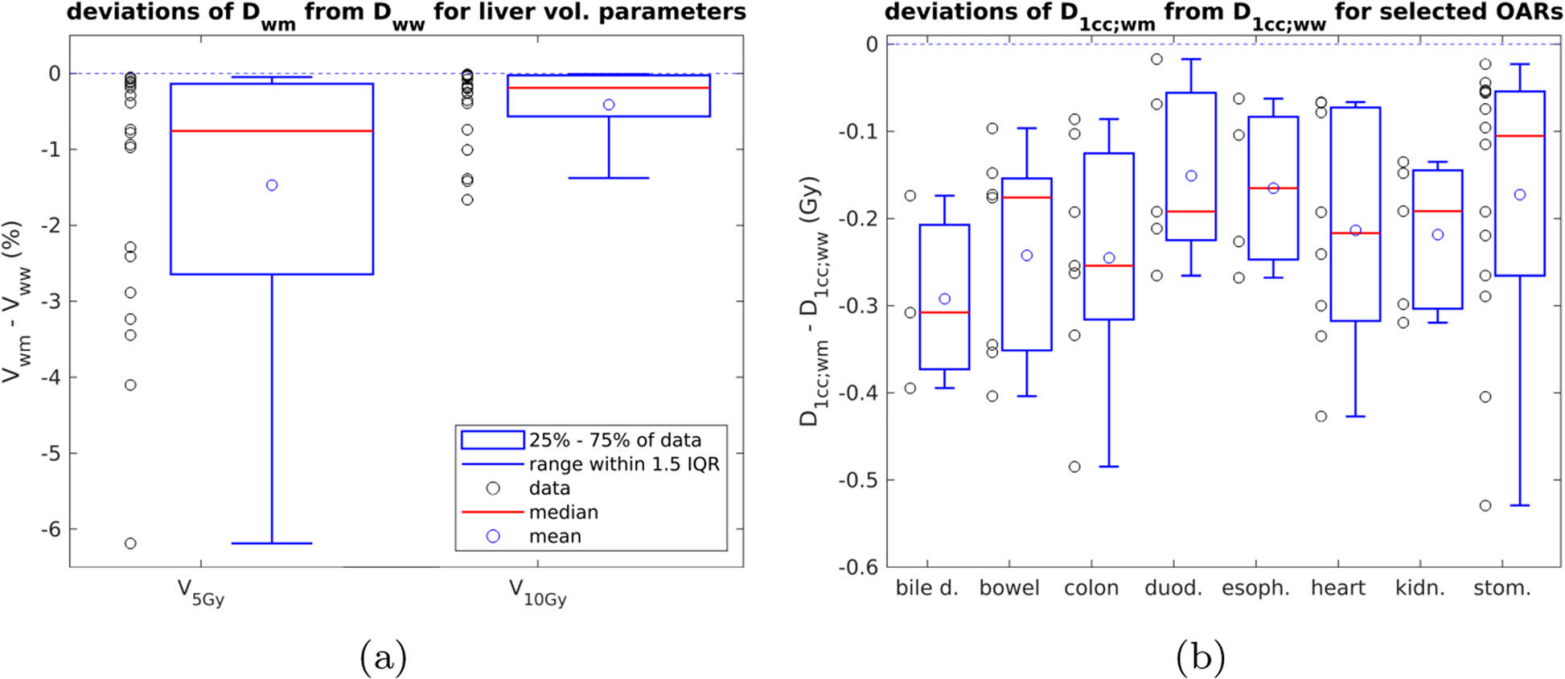
Deviations of DVH parameters calculated with *D*_w,m_ from *D*_w,w_ for liver and OARs. IQR stands for interquartile range, which is equal to the spread of the central 50% of the data. **a** Deviations for liver parameters. **b** Deviations for selected OARs

As an assessment of the influence of TG-186 on DVH parameters for the liver, we report *D*_w,w_-calculated parameters as well as the absolute difference between *D*_w,m_- and *D*_w,w_-calculated parameters for *V*_5Gy_ and *V*_10Gy_ shown in Table 3 and Fig. 1a. The median of *D*_w,w_ was well within clinical tolerance levels, the maximum values exceeded tolerances by a few percent for both *V*_5Gy_ and *V*_10Gy_. The values of *V*_5Gy_ and *V*_10Gy_ showed a very high spread, ranging from a few percent to values above the tolerance limit. The deviations were most pronounced for *V*_5Gy_, ranging from -0.05% to -6.2%. *V*_10Gy_-deviations ranged from -0.01% to -1.7%. The median deviation was -0.8% for *V*_5Gy_ and -0.2% for *V*_10Gy_.

**Table 3:** Comparison of *D*_w,w_ and *D*_w,m_ for liver DVH parameters

Parameter (%)	*D*_w,w_			*D*_w,m_−*D*_w,w_			clin. tol.
	Median	Min	Max	Median	Min	Max	
*V*_5Gy_	16	2	72	-0.8	-6.2	-0.05	67
*V*_10Gy_	7	1	38	-0.2	-1.7	-0.01	33

The total number of livers, equal to the total number of cases, was 20. All parameters are given in percentage of the total liver volume and correspond therefore to absolute deviations, not relative to the value of the DVH parameter. The last column shows clinical tolerance levels for the liver

For the case with highest deviation (case ii, -6.2%) the *V*_5Gy_-parameter calculated with *D*_w,w_ was also the maximum value of all cases, 72%. It exceeded the tolerance of 67%. With the reduction of -6.2%, the *D*_w,m_-calculated value was just below the tolerance limit (66%). The *V*_10Gy_-parameter was also quite high (26%), but did not exceed the tolerance of 33%. For this value, the deviation was -1.7%, which is also the deviation of highest magnitude over all cases for the *V*_10Gy_-parameter.

**Fig. 2:**
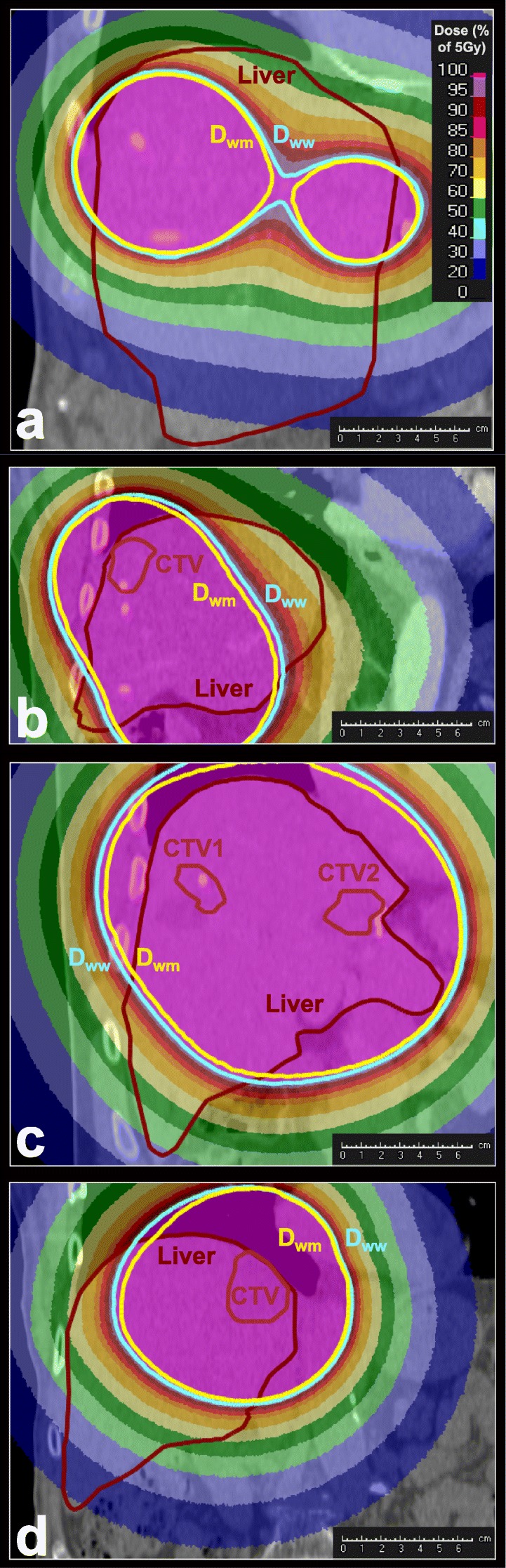
Dose distributions of *D*_w,w_ for coronal slices of 4 different cases. The dose profile is normalised so that 100% dose equals 5 Gy. Overlaid are the contours of the 5-Gy-isodose lines of *D*_w,w_ (light blue) and *D*_w,m_ (yellow). Additional contours show the liver (dark red) and one or more CTVs (red). This figure focuses on the analysis of the *V*_5Gy_-parameter and is not suitable for evaluation of target coverage, since the CTVs are not always shown. **a** case i. **b** case ii. **c** case iv. **d** case xix

Figure 2 shows the dose distributions for this case and three other cases, calculated with *D*_w,w_. For case i, the reduction of the *V*_5Gy_ when comparing *D*_w,m_ to *D*_w,w_ is clearly visible in the region between two CTVs. The liver is only partly covered by the 5-Gy-isodose line. For case ii, the volume of the *D*_w,m_-calculated 5-Gy-isodose is notably smaller than the *D*_w,w_-calculated volume. Both cover a large part of the liver. This can also be seen in case iv, where *D*_w,w_ and *D*_w,m_ show a deviation close to the body surface. A large part of the liver is enclosed by the 5-Gy-isodose lines in this case. For case xix, the *D*_w,w_- and *D*_w,m_-isodose lines are quite close together, except at lung-tissue interface regions.

### OAR dose

For the OARs we report the difference between *D*_w,m_ and *D*_w,w_ for *D*_1cc_ of each OAR type (see Table 4 and Fig. 1b). The differences in *D*_1cc_ ranged from -0.02 Gy to -0.5 Gy. The median differences were of similar magnitude for all types of OAR. The highest deviations were found for colon (-0.5 Gy, -4.8% relative to *D*_w,w_-value) and stomach (-0.5 Gy, -3.7% relative to *D*_w,w_-value) of case iv.

**Table 4:** Comparison of *D*_w,w_ and *D*_w,m_ for *D*_1cc_ of the selected OARs

OAR	Count	*D*_1cc;w,w_ (Gy)			*D*_1cc;w,m_−*D*_1cc;w,w_ (Gy)			clin. tol. (Gy)
		Median	Min	Max	Median	Min	Max	
Bile duct	3	12.9	10.4	16.5	-0.3	-0.4	-0.2	21
Bowel	7	8.0	4.8	13.2	-0.2	-0.4	-0.1	15
Colon	7	5.7	2.6	12.0	-0.3	-0.5	-0.1	20
Duodenum	5	7.1	1.4	7.8	-0.2	-0.3	-0.02	12
Esophagus	4	5.3	3.0	7.7	-0.2	-0.3	-0.1	12
Gall bladder	1	11.5	-	-	-0.3	-	-	20
Heart	8	9.0	2.7	13.5	-0.2	-0.4	-0.1	22
Kidney	5	17.4	8.7	22.5	-0.2	-0.3	-0.1	-
Stomach	14	8.0	1.6	14.2	-0.1	-0.5	-0.02	12

The list of OARs for each case can be found in Table 1. The number of cases containing each OAR is listed in column 2. The last column shows clinical tolerance levels for the OARs. For kidney, the *D*_1cc_ is not a parameter that is used in clinical practice, therefore there is no tolerance level for this organ. All parameters are given in Gy

The median *D*_w,w_-values were within clinical tolerance limits for most OAR types. There was one case where the *D*_1cc_ of the stomach exceeded the limit of 12 Gy both when calculated with *D*_w,w_ (14.2 Gy) and *D*_w,m_ (13.7 Gy).

### Target coverage

**Fig. 3:**
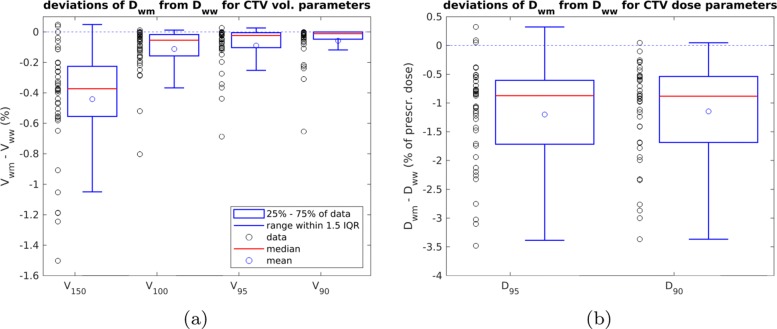
Deviations of DVH parameters used to analyse target coverage. IQR stands for interquartile range, which is equal to the spread of the central 50% of the data. **a** Deviations of CTV volume parameters. **b** Deviations of CTV dose parameters

Table 5 shows the values of the CTV DVH parameters, calculated for *D*_w,w_, as well as deviations of *D*_w,m_-calculated values from the latter. The deviations are also visualized in Fig. 3a and b. We observed little change for *V*_100_, *V*_95_ and *V*_90_ when comparing *D*_w,m_ and *D*_w,w_, with the median deviation being -0.05% for *V*_100_, -0.02% for *V*_95_ and -0.01% for *V*_90_. The most important deviation of these parameters was -0.8%. Figure 3a shows that most of the values are concentrated in a range of very low deviation with only a few exceptions. *V*_150_ showed a higher deviation of *D*_w,m_ from *D*_w,w_ with a median of -0.4% and a maximum deviation in absolute terms of -1.5%. *V*_150_-deviations were more widely spread.

**Table 5:** Comparison of *D*_w,w_ and *D*_w,m_ for CTV DVH parameters

Parameter (%)	*D*_w,w_			*D*_w,m_−*D*_w,w_		
	Median	Min	Max	Median	Min	Max
*V*_150_	85	67	100	-0.4	-1.5	0.05
*V*_100_	100	91	100	-0.05	-0.8	0.01
*V*_95_	100	93	100	-0.02	-0.7	0.03
*V*_90_	100	95	100	-0.01	-0.7	0
*D*_95_	123	90	256	-0.9	-3.5	0.3
*D*_90_	139	101	302	-0.9	-3.4	0.05

The number of treated lesions for each case can be found in Table 1. The total number of treated lesions over all cases was 41. All volume parameters are given in percentage of the total CTV volume, all dose parameters in percentage of the prescribed dose

Concerning the dose parameters *D*_95_ and *D*_90_, the deviations from TG-43U1 had a median value of -0.9% of the prescribed dose for *D*_95_ and *D*_90_. The deviations of largest magnitude were -3.5% for *D*_95_ and -3.4% of the prescribed dose for *D*_90_. For *D*_95_, the maximum deviation was found to be 0.3% of the prescribed dose. Since this value corresponds to 0.07 Gy in absolute units, it is considered negligible. The maximum values of *D*_95_ (256% of the prescribed dose) and *D*_90_ (302% of the prescribed dose) corresponded to a case where two CTVs were located very close to one another which resulted in an accumulation of dose in that region.

## Discussion

Our calculations found TG-186-calculated DVH-parameters to be in general lower than TG-43U1-calculated parameters. This is in good agreement with the results of other groups who reported an overestimation of TG-43U1 in comparison to TG-186 for soft tissue in other tumour entities [4,6,8,10]. For breast cancer cases, Thrower et al. compared TG-43 to *D*_m,m_ and reported relative percentage changes of 1.2% for *V*_100_, 0.86% for *V*_95_ and 0.47% for *V*_90_. Deviations in *D*_1cc_ were 3.1% for the rib, 2.5% for the skin and 3.1% for the lung [4]. Fotina et al. found a relative percentage change in target coverage of about 2% for breast, esophageal and gynecologic cases, comparing *D*_w,m_ to TG-43 [6]. Peppa et al. reported changes when comparing *D*_m,m_ to TG-43 of up to 2% relative to the prescription dose for head and neck cancer. Regarding target coverage, relative deviations were as high as 9.1% for *V*_200_ and 5.2% for *V*_150_ [8]. For cervix cancer patients, Jacob et al. compared TG-43 to *D*_m,m_ and reported changes in target coverage parameter *D*_90_ of 2.6%, with similar values for *D*_95_ and *D*_100_. They found an average relative change of 2.8% in *D*_1cc_ for rectum, bladder and sigmoid [10].

We decided to focus on *D*_w,m_-values in the comparison to *D*_w,w_, since *D*_m,m_-values were very similar to *D*_w,m_. This was expected for soft tissues in the range of ^192^Ir-energies [1] and will therefore not be further addressed in this study.

### Liver dose

The deviations of the *V*_5Gy_- and *V*_10Gy_-parameter are of a magnitude that may be clinically relevant, especially *V*_5Gy_ shows large differences between *D*_w,w_ and *D*_w,m_. Since deviations differed strongly from case to case, we tried to find a case-specific feature determining the magnitude of deviation. As a first attempt, we looked at the dependence of deviations on the *V*_5Gy_-parameter. This volume was usually very large compared to other volume parameters (*V*_95_, *V*_90_) resulting in a large travel distance for photons through the liver. Since liver density is higher than water density, photons do not travel as far as in water, and the *D*_w,m_-isodose line is pulled back in comparison to *D*_w,w_. This effect should be more pronounced for higher values of the *V*_5Gy_-parameter due to an increase in photon travel distance.

**Fig. 4:**
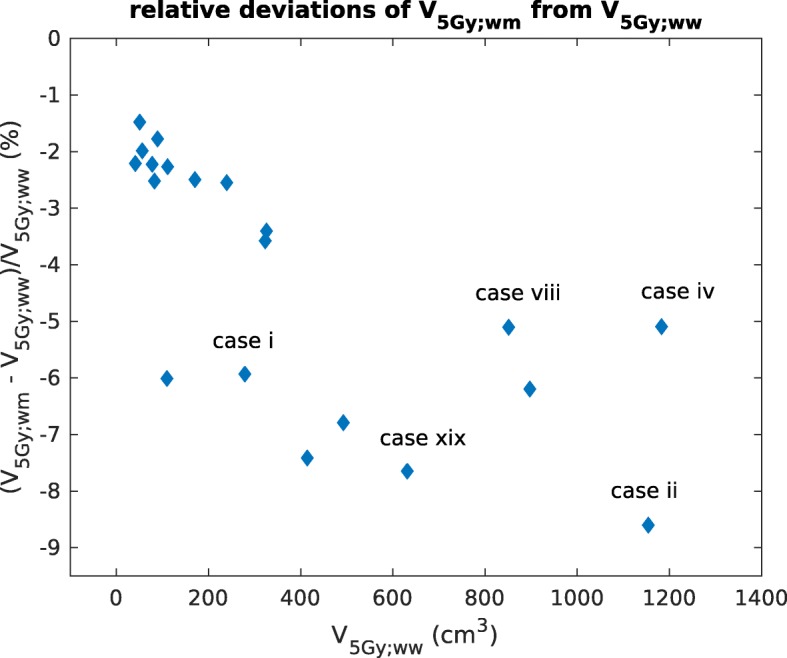
Relative deviations of the *V*_5Gy_-parameter from TG-43U1, plotted over the respective TG-43U1 parameter value in c*m*^3^. The labels correspond to the cases shown in Fig. 2

To find correlations of the deviations of *V*_5Gy_ and the *V*_5Gy_-parameter itself, we plotted in Fig. 4 the relative deviations $\frac {V_{\mathrm {5Gy;w,m}} - V_{\mathrm {5Gy;w,w}}}{V_{\mathrm {5Gy;w,w}}}$ over *V*_5Gy;w,w_ in c*m*^3^. Since absolute deviations are expected to scale with *V*_5Gy;w,w_, relative deviations are shown in this plot. Furthermore, since total liver volumes can vary a lot among patients, we show the *V*_5Gy_-parameter in absolute units of c*m*^3^ instead of percentages.

Due to a number of factors such as the large spread of prescription doses, the variation in number and size of CTVs and the location of CTVs, there is also a large spread in the values of the *V*_5Gy_-parameter. The plot shows that cases with a larger *V*_5Gy;w,w_ tend to have relative deviations of larger magnitude. This tendency also explains why deviations of the *V*_10Gy_-parameter, which is usually much smaller than the *V*_5Gy_-parameter, are also smaller in general. Cases marked in the plot correspond to those shown in Fig. 2. The effect of a higher *V*_5Gy_-volume on the separation of the two isodose lines is clearly visible when comparing case i (Fig. 2a) to the other cases (Fig. 2b-d), neglecting for case i the region between the two CTVs where the separation of the isodose lines is larger.

Other groups also suggested a correlation between deviations from TG-43U1 and photon travel distance path. Thrower et al. and Zourari et al. found that deviations increased with increasing distance from dwell positions [4,5] for breast cases, although in that case this was mostly due to lung heterogeneities.

Apart from a pure dependency on photon travel distance we also found other possible influences of the magnitude of the *V*_5Gy_-parameter deviation. An example is case i: if we look at Fig. 2a, we see that this case had multiple treated CTVs (3 in total) and the dose in the overlap region between two CTVs was very close to 5 Gy. Since this region is fully enclosed by the liver, it has a high influence on the *V*_5Gy_-parameter deviation. This example shows that the number of source positions or CTVs per case may play a role in the magnitude of deviations of the *V*_5Gy_-parameter, given that the dose between CTVs is in the range of 5 Gy and the CTVs are not too close together.

Another influence on the *V*_5Gy_-parameter is shown in case iv (Fig. 2c). We can see that the *V*_5Gy_-volume is very large and only a smaller part of the liver is actually influenced by the deviations of *D*_w,w_ and *D*_w,m_. This example demonstrates that not only the magnitude of the *V*_5Gy_-parameter has an influence on the magnitude of the deviation from TG-43U1, but that also the location of the 5-Gy-isodose lines and therefore of the CTVs is important. If the isodose lines are located mostly inside of the liver, deviations will be much more pronounced than in cases like case iv, where most of the isodose lines are outside of the liver.

As can be seen in cases ii, iv and xix (Fig. 2b, c, d), proximity to lung, body surface or bone also has an influence on the 5-Gy-isodose. However, since sources are always placed inside the liver and only the percentage of the *V*_5Gy_-volume that covers the liver is clinically relevant, these factors do not have a strong influence on the parameter itself. Only in cases where the 5-Gy-isodose line lies within the liver and at the same time close to a lung-tissue interface region (see Fig. 2d), loss of backscatter due to less photon attenuation in low-density tissue and therefore a reduction in *D*_w,m_-dose is visible.

The data analysed in this study suggests that replacing TG-43U1 with TG-186 may be of clinical relevance if we look at typical parameters for the assessment of liver toxicity. There were a few cases where liver parameters were close to the tolerance limit. TG-186-calculated parameters showed a reduction in these parameters of up to 6.2% of the absolute liver volume. This result is important in clinical decision making regarding cases where a sufficient local tumour control can only be achieved by giving a relatively high dose to a large part of the liver. The wide spread of relative deviations for the *V*_5Gy_-parameter from a few percent up to almost -9% (see Fig. 4) shows that there is a variety between cases that is not taken into account when only TG-43U1 is used for dose calculations. Studies have been performed on the tolerance of liver tissue to brachytherapy using radiation-induced liver damage (RILD) as a biological endpoint [39]. Our findings support a reassessment of current tolerance limits when computing dose following TG-186.

### OAR dose

For the OARs, the deviations in absolute terms were small. There was one case (case iv) where the difference was -0.5 Gy for colon and for stomach. These organs were analysed in the CT images and a possible reason for increased deviations could be a larger distance from the brachytherapy source and therefore a reduction in dose because photons are travelling through liver tissue of higher density. However, this reduction is too small to have an impact on clinical treatment planning dose.

### Target coverage

CTV volume parameters *V*_100_, *V*_95_ and *V*_90_ show very small deviations from *D*_w,w_ for most of the cases. This is primarily due to a very good coverage of the CTVs: For *V*_100_, *V*_95_ and *V*_90_ the median value of *D*_w,w_ was 100%. Therefore, any deviations from TG-43U1 can hardly be seen in these parameters. *V*_150_ has a median value of 85% and shows higher deviations from TG-43U1. This can be explained by the fact that this parameter was always located in a region of higher gradient of the DVH, where small deviations cause a major change of parameter value. These results agree with the findings of other groups who also reported an increase in deviations for *V*_150_ compared to the other volume parameters [5,8]. However, *V*_150_ and *V*_100_ are of little clinical relevance in brachytherapy for liver metastases, because there is no demand for a homogeneous dose profile in the target volumes. These parameters were mainly included for better comparison to other brachytherapy treatment sites such as breast, where they play an important role in determining plan quality.

The dose parameters *D*_95_ and *D*_90_ show slightly larger deviations than the volume parameters when comparing the percentage values. This can be explained by the fact that these values are located in the gradient of the DVH curve, and no longer in the plateau like most of the volume parameters. Still, these deviations are all well below 1 Gy and will therefore have no major influence on the target coverage.

In this study we observed that the location and number of dwell positions as well as the liver volume are important parameters that influence the magnitude of deviations between TG-43U1 and TG-186, thus it may be interesting to systematically investigate their combined effect. For cases where doses to the whole liver are close to tolerance limits and where TG-186 dose is notably reduced compared to dose calculated by TG-43U1, a reassessment of tolerance doses may be beneficial. Further studies are needed to investigate this issue, since model-based dose calculations are not yet used as a standard in clinical practice.

The results of this study are limited by the tissue segmentation which only included 5 tissues. Furthermore, there was a high variety in number, size and location of CTVs, number and type of OARs and prescription doses, which makes a quantitative comparison between cases difficult.

Photon propagation through medium leads to modifications in the energy spectrum which increase with larger distance from the source. For ^192^Ir, modifications were found to be of a relevant magnitude in a previous study [31]. This can lead to errors in the conversion of *D*_w,m_ to *D*_m,m_ and vice versa. Since both of these properties were calculated on the fly in MCNP instead of employing any conversion factors, our results are not affected by these errors.

Since the liver is subject to a lot of intrafractional motion and treatment times can take up to 60 min [40], performing dose calculations on 4DCT data would be highly useful for estimating additional dose calculation errors due to motion. Respiratory motion has proven to have an impact on stereotactic ablative radiotherapy (SABR) plans for the treatment of hepatocellular carcinoma [41] due to the high magnitude of liver motion, which can exceed 5 mm even during usage of abdominal compression [42] and is different for different liver segments [43]. Another area of interest would be cases where the lung or parts of the bone, e.g. the thoracic wall, are considered to be OARs. Due to the highly different density compared to water these organs would supposedly be more sensitive to deviations of TG-186 from TG-43U1.

## Conclusion

This study is to our knowledge the first one comparing TG-43U1 to TG-186 for the interstitial ^192^Ir HDR brachytherapy treatment of liver metastases and hepatocellular carcinoma. We found a general overestimation of DVH parameters by TG-43U1 compared to TG-186, especially regarding the liver *V*_5Gy_-parameter, of up to 6.2% of liver volume. Deviations from TG-43U1 regarding dose to OARs and target coverage were all well below 1 Gy. We conclude that there may be a clinical benefit of reporting TG-186 alongside TG-43U1 for cases where differences between the calculation methods are high and doses to the whole liver are close to tolerance limits.

## Appendix

**Table 6:** Comparison of *D*_w,w_ and *D*_w,m_ as well as *D*_w,w_ and *D*_m,m_ for liver DVH parameters

Parameter (%)	*D*_w,w_	*D*_w,m_−*D*_w,w_			*D*_m,m_−*D*_w,w_		
	Median	Median	Min	Max	Median	Min	Max
*V*_5Gy_	16	-0.8	-6.2	-0.05	-0.9	-6.7	-0.07
*V*_10Gy_	7	-0.2	-1.7	-0.01	-0.2	-1.9	-0.02

The total number of livers, equal to the total number of cases, was 20. All parameters are given in percentage of the total liver volume

**Table 7:** Comparison of *D*_w,w_ and *D*_w,m_ as well as *D*_w,w_ and *D*_m,m_ for *D*_1cc_ of the selected OARs

OAR	Count	*D*_1cc;w,w_ (Gy)	*D*_1cc;w,m_−*D*_1cc;w,w_ (Gy)			*D*_1cc;m,m_−*D*_1cc;w,w_ (Gy)		
		Median	Median	Min	Max	Median	Min	Max
Bile duct	3	12.9	-0.3	-0.4	-0.2	-0.4	-0.5	-0.3
Bowel	7	8.0	-0.2	-0.4	-0.1	-0.3	-0.5	-0.1
Colon	7	5.7	-0.3	-0.5	-0.09	-0.3	-0.9	-0.1
Duodenum	5	7.1	-0.2	-0.3	-0.02	-0.2	-0.3	-0.04
Esophagus	4	5.3	-0.2	-0.3	-0.06	-0.2	-0.3	-0.1
Gall bladder	1	11.5	-0.3	-	-	-0.4	-	-
Heart	8	9.0	-0.2	-0.4	-0.07	-0.3	-0.6	-0.1
Kidney	5	17.4	-0.2	-0.3	-0.1	-0.3	-0.5	-0.2
Stomach	14	8.0	-0.1	-0.5	-0.02	-0.2	-0.6	-0.07

The list of OARs for each case can be found in Table 1. The number of cases containing each OAR is listed in column 2. All parameters are given in Gy

**Table 8:** Comparison of *D*_w,w_ and *D*_w,m_ as well as *D*_w,w_ and *D*_m,m_ for CTV DVH parameters

Parameter (%)	*D*_w,w_	*D*_w,m_−*D*_w,w_			*D*_m,m_−*D*_w,w_		
	Median	Median	Min	Max	Median	Min	Max
*V*_150_	85	-0.4	-1.5	0.05	-0.8	-2.0	0
*V*_100_	100	-0.05	-0.8	0.01	-0.1	-1.1	0
*V*_95_	100	-0.02	-0.7	0.03	-0.06	-1.0	0
*V*_90_	100	-0.01	-0.7	0	-0.02	-0.9	0
*D*_95_	123	-0.9	-3.5	0.3	-1.9	-4.4	-0.8
*D*_90_	139	-0.9	-3.4	0.05	-1.8	-4.4	-1.0

The number of treated lesions for each case can be found in Table 1. The total number of treated lesions over all cases was 41. All volume parameters are given in percentage of the total CTV volume, all dose parameters in percentage of the prescribed dose

## Supplementary information

**Additional file 1** Supplementary material.

## Data Availability

The datasets supporting the conclusions of this article are included within the article.

## Abbreviations

4DCT: Four-dimensional computed tomography
AAPM: American Association of Physicists in Medicine
CTV: Clinical target volume
CT: Computed tomography
*D*_1cc_: Dose received by 1 cubic centimeter
*D*_95_: Dose received by 95% of the volume
*D*_90_: Dose received by 90% of the volume
DVH: Dose volume histogram
*D*_m,m_: Dose calculated and scored in medium
*D*_w,m_: Dose calculated in medium and scored in water
*D*_w,w_: Dose calculated and scored in water
Gy: Gray
HCC: Hepatocellular carcinoma
HDR: High dose rate
HU: Hounsfield Unit
IQR: Interquartile range
Ir: Iridium
mCRC: Metastatic colorectal cancer
MBDC: Model-based dose calculation
MC: Monte Carlo
MCNP: Monte Carlo n-Particle
OAR: Organ at risk
PTV: Planning target volume
RFA: Radiofrequency ablation
RILD: Radiation-induced liver damage
SABR: Stereotactic ablative radiotherapy
SBRT: Stereotactic body radiation therapy
TACE: Transarterial chemoembolization
TG: Task group
TPS: Treatment planning system
*V*_5Gy_: Volume receiving at least 5 Gy
*V*_10Gy_: Volume receiving at least 10 Gy
*V*_150_: Volume receiving at least 150% of the prescribed dose
*V*_100_: Volume receiving at least 100% of the prescribed dose
*V*_95_: Volume receiving at least 95% of the prescribed dose
*V*_90_: Volume receiving at least 90% of the prescribed dose

## References

[1] Beaulieu L, Carlsson Tedgren Å, Carrier JF, Davis SD, Mourtada F, Rivard MJ, Thomson RM, Verhaegen F, Wareing TA, Williamson JF (2012). Report of the Task Group 186 on model-based dose calculation methods in brachytherapy beyond the TG-43 formalism: Current status and recommendations for clinical implementation. Med Phys.

[2] Rivard MJ, Coursey BM, DeWerd LA, Hanson WF, Saiful Huq M, Ibbott GS, Mitch MG, Nath R, Williamson JF (2004). Update of AAPM Task Group No. 43 Report: A revised AAPM protocol for brachytherapy dose calculations. Med Phys.

[3] Afsharpour H, D’Amours M, Coté B, Carrier J-F, Verhaegen F, Beaulieu L (2008). A Monte Carlo study on the effect of seed design on the interseed attenuation in permanent prostate implants. Med Phys.

[4] Thrower SL, Shaitelman SF, Bloom E, Salehpour M, Gifford K (2016). Comparison of Dose Distributions With TG-43 and Collapsed Cone Convolution Algorithms Applied to Accelerated Partial Breast Irradiation Patient Plans. Int J Radiat Oncol Biol Phys.

[5] Zourari K, Major T, Herein A, Peppa V, Polgár C, Papagiannis P (2015). A retrospective dosimetric comparison of TG43 and a commercially available MBDCA for an APBI brachytherapy patient cohort. Phys Medica.

[6] Fotina I, Zourari K, Lahanas V, Pantelis E, Papagiannis P (2018). A comparative assessment of inhomogeneity and finite patient dimension effects in 60Co and 192Ir high-dose-rate brachytherapy. J Contemp Brachytherapy.

[7] White SA, Landry G, van Gils F, Verhaegen F, Reniers B (2012). Influence of trace elements in human tissue in low-energy photon brachytherapy dosimetry. Phys Med Biol.

[8] Peppa V, Pappas E, Major T, Takácsi-Nagy Z, Pantelis E, Papagiannis P (2016). On the impact of improved dosimetric accuracy on head and neck high dose rate brachytherapy. Radiother Oncol.

[9] Abe K, Kadoya N, Sato S, Hashimoto S, Nakajima Y, Miyasaka Y, Ito K, Umezawa R, Yamamoto T, Takahashi N, Takeda K, Jingu K (2018). Impact of a commercially available model-based dose calculation algorithm on treatment planning of high-dose-rate brachytherapy in patients with cervical cancer. J Radiat Res.

[10] Jacob D, Lamberto M, DeSouza Lawrence L, Mourtada F (2017). Clinical transition to model-based dose calculation algorithm: A retrospective analysis of high-dose-rate tandem and ring brachytherapy of the cervix. Brachytherapy.

[11] Terribilini D, Vitzthum V, Volken W, Frei D, Loessl K, van Veelen B, Manser P, Fix MK (2017). Performance evaluation of a collapsed cone dose calculation algorithm for HDR Ir-192 of APBI treatments. Med Phys.

[12] Ahnesjö A (1989). Collapsed cone convolution of radiant energy for photon dose calculation in heterogeneous media. Med Phys.

[13] Ma Yunzhi, Lacroix Fréderic, Lavallée Marie-Claude, Beaulieu Luc (2015). Validation of the Oncentra Brachy Advanced Collapsed cone Engine for a commercial 192Ir source using heterogeneous geometries. Brachytherapy.

[14] Hofbauer J, Kirisits C, Resch A, Xu Y, Sturdza A, Pötter R, Nesvacil N (2016). Impact of heterogeneity-corrected dose calculation using a grid-based Boltzmann solver on breast and cervix cancer brachytherapy. J Contemp Brachytherapy.

[15] Vassiliev O, Wareing T, McGhee J, Failla G, Salehpour M, Mourtada F (2010). Validation of a new grid-based Boltzmann equation solver for dose calculation in radiotherapy with photon beams. Phys Med Biol.

[16] Rogers DWO (2006). Fifty years of Monte Carlo simulations for medical physics. Phys Med Biol.

[17] Krause F, Risske F, Bohn S, Delaperriere M, Dunst J, Siebert F-A (2018). End-to-end test for computed tomography-based high-dose-rate brachytherapy. J Contemp Brachytherapy.

[18] Ricke J, Wust P (2011). Computed Tomography-Guided Brachytherapy for Liver Cancer. Semin Radiat Oncol.

[19] Kieszko D, Cisek P, Kordzińska-Cisek I, Grzybowska-Szatkowska L (2018). Treatment of hepatic metastases with computed tomography-guided interstitial brachytherapy. Oncol Lett.

[20] Gerum S, Heinz C, Belka C, Walter F, Paprottka P, De Toni EN, Roeder F. Stereotactic body radiation therapy (SBRT) in patients with hepatocellular carcinoma and oligometastatic liver disease. Radiat Oncol. 2018; 13(1). 10.1186/s13014-018-1048-4. 10.1186/s13014-018-1048-4 PMC5975506 29843752

[21] Hass P, Mohnike K, Kropf S, Brunner TB, Walke M, Albers D, Petersen C, Damm R, Walter F, Ricke J, Powerski M, Corradini S (2019). Comparative analysis between interstitial brachytherapy and stereotactic body irradiation for local ablation in liver malignancies. Brachytherapy.

[22] Scorsetti M, Comito T, Clerici E, Franzese C, Tozzi A, Iftode C, Di Brina L, Navarria P, Mancosu P, Reggiori G, Fogliata A, Tomatis S, Torzilli G, Cozzi L (2018). Phase II trial on SBRT for unresectable liver metastases: long-term outcome and prognostic factors of survival after 5 years of follow-up. Radiat Oncol.

[23] Mahadevan A, Blanck O, Lanciano R, Peddada A, Sundararaman S, D’Ambrosio D, Sharma S, Perry D, Kolker J, Davis J (2018). Stereotactic Body Radiotherapy (SBRT) for liver metastasis – clinical outcomes from the international multi-institutional RSSearch® Patient Registry. Radiat Oncol.

[24] Gkika E, Schultheiss M, Bettinger D, Maruschke L, Neeff HP, Schulenburg M, Adebahr S, Kirste S, Nestle U, Thimme R, Grosu A-L, Brunner TB (2017). Excellent local control and tolerance profile after stereotactic body radiotherapy of advanced hepatocellular carcinoma. Radiat Oncol.

[25] Mohnike K, Steffen IG, Seidensticker M, Hass P, Damm R, Peters N, Seidensticker R, Schütte K, Arend J, Bornschein J, Streitparth T, Wybranski C, Wieners G, Stübs P, Malfertheiner P, Pech M, Ricke J (2019). Radioablation by Image-Guided (HDR) Brachytherapy and Transarterial Chemoembolization in Hepatocellular Carcinoma: A Randomized Phase II Trial. Cardiovasc Intervent Radiol.

[26] Shiba S, Shibuya K, Katoh H, Kaminuma T, Miyazaki M, Kakizaki S, Shirabe K, Ohno T, Nakano T (2019). A comparison of carbon ion radiotherapy and transarterial chemoembolization treatment outcomes for single hepatocellular carcinoma: a propensity score matching study. Radiat Oncol.

[27] Schnapauff D, Collettini F, Steffen I, Wieners G, Hamm B, Gebauer B, Maurer MH (2016). Activity-based cost analysis of hepatic tumor ablation using CT-guided high-dose rate brachytherapy or CT-guided radiofrequency ablation in hepatocellular carcinoma. Radiat Oncol.

[28] Fonseca GP, Reniers B, Landry G, White S, Bellezzo M, Antunes PCG, de Sales CP, Welteman E, Yoriyaz H, Verhaegen F (2014). A medical image-based graphical platform-Features, applications and relevance for brachytherapy. Brachytherapy.

[29] Bretschneider T, Ricke J, Gebauer B, Streitparth F (2016). Image-guided high-dose-rate brachytherapy of malignancies in various inner organs-technique, indications, and perspectives. J Contemp Brachytherapy.

[30] Goorley T, James M, Booth T, Brown F, Bull J, Cox LJ, Durkee J, Elson J, Fensin M, Forster RA, Hendricks J, Hughes HG, Johns R, Kiedrowski B, Martz R, Mashnik S, McKinney G, Pelowitz D, Prael R, Sweezy J, Waters L, Wilcox T, Zukaitis T (2012). Initial MCNP6 release overview. Nucl Technol.

[31] Fonseca GP, Tedgren Å. C., Reniers B, Nilsson J, Persson M, Yoriyaz H, Verhaegen F (2015). Dose specification for 192Ir high dose rate brachytherapy in terms of dose-to-water-in-medium and dose-to-medium-in-medium. Phys Med Biol.

[32] Ballester F, Granero D, Ṕrez-Calatayud J, Melhus CS, Rivard MJ (2009). Evaluation of high-energy brachytherapy source electronic disequilibrium and dose from emitted electrons. Med Phys.

[33] Wang R, Li XA (2002). Dose characterization in the near-source region for two high dose rate brachytherapy sources. Med Phys.

[34] Berger MJ, Hubbell JH, Seltzer SM, Chang J, Coursey JS, Sukumar R, Zucker DS, Olsen K. XCOM: Photon Cross Section Database (Version 1.3). 2005. https://www.nist.gov/pml/xcom-photon-cross-sections-database.

[35] Baglin Coral M. (2012). Nuclear Data Sheets for A = 192. Nuclear Data Sheets.

[36] Taylor REP, Rogers DWO (2008). EGSnrc Monte Carlo calculated dosimetry parameters for 192Ir and 169Yb brachytherapy sources. Med Phys.

[37] Ma Y, Vijande J, Ballester F, Tedgren Å. C., Granero D, Haworth A, Mourtada F, Fonseca GP, Zourari K, Papagiannis P, Rivard MJ, Siebert FA, Sloboda RS, Smith R, Chamberland MJP, Thomson RM, Verhaegen F, Beaulieu L (2017). A generic TG-186 shielded applicator for commissioning model-based dose calculation algorithms for high-dose-rate 192Ir brachytherapy:. Med Phys.

[38] Ballester Facundo, Carlsson Tedgren Åsa, Granero Domingo, Haworth Annette, Mourtada Firas, Fonseca Gabriel Paiva, Zourari Kyveli, Papagiannis Panagiotis, Rivard Mark J., Siebert Frank-André, Sloboda Ron S., Smith Ryan L., Thomson Rowan M., Verhaegen Frank, Vijande Javier, Ma Yunzhi, Beaulieu Luc (2015). A generic high-dose rate192Ir brachytherapy source for evaluation of model-based dose calculations beyond the TG-43 formalism. Medical Physics.

[39] Seidensticker M, Burak M, Kalinski T, Garlipp B, Koelble K, Wust P, Antweiler K, Seidensticker R, Mohnicke K, Pech M, Ricke J (2015). Radiation-Induced Liver Damage: Correlation of Histopathology with Hepatobiliary Magnetic Resonance Imaging, a Feasibility Study. Cardiovasc Intervent Radiol.

[40] Lüdemann L, Wybranski C, Seidensticker M, Mohnike K, Kropf S, Wust P, Ricke J. In vivo assessment of catheter positioning accuracy and prolonged irradiation time on liver tolerance dose after single-fraction 192Ir high-dose-rate brachytherapy. Radiat Oncol. 2011; 6(1). 10.1186/1748-717X-6-107. 10.1186/1748-717X-6-107 PMC3179944 21892943

[41] Gargett M, Haddad C, Kneebone A, Booth JT, Hardcastle N. Clinical impact of removing respiratory motion during liver SABR. Radiat Oncol. 2019; 14(1). 10.1186/s13014-019-1300-6. 10.1186/s13014-019-1300-6 PMC6547575 31159840

[42] Hu Y, Zhou YK, Chen YX, Zeng ZC. Magnitude and influencing factors of respiration-induced liver motion during abdominal compression in patients with intrahepatic tumors. Radiat Oncol. 2017; 12(1). 10.1186/s13014-016-0762-z. 10.1186/s13014-016-0762-z PMC5223487 28073377

[43] Tsai YL, Wu CJ, Shaw S, Yu PC, Nien HH, Lui LT (2018). Quantitative analysis of respiration-induced motion of each liver segment with helical computed tomography and 4-dimensional computed tomography. Radiat Oncol.

