# Supplementary material for: The dosimetric impact of replacing the TG-43 algorithm by model based dose calculation for liver brachytherapy
Source: Radiat Oncol. 2020 Mar 9;15:60. doi: 10.1186/s13014-020-01492-9 (PMC7063719; doi:10.1186/s13014-020-01492-9)
Supplement: Supplementary file 1 — Additional file 1 Supplementary material. [file 13014_2020_1492_MOESM1_ESM.pdf]

Supplementary Material

Table S1 Comparison of $D_{\mathrm{w,w}}$ and $D_{\mathrm{w,m}}$ as well as $D_{\mathrm{w,w}}$ and $D_{\mathrm{m,m}}$ for liver DVH parameters. The total number of livers, equal to the total number of cases, was 20. The $D_{\mathrm{w,w}}$-value is given in percentage of the total liver volume, the deviations from $D_{\mathrm{w,w}}$ in percentage of the $D_{\mathrm{w,w}}$-value.

parameter (%)	$D_{\mathrm{w,w}}$	$\frac{D_{\mathrm{w,m}}-D_{\mathrm{w,w}}}{D_{\mathrm{w,w}}}$			$\frac{D_{\mathrm{m,m}}-D_{\mathrm{w,w}}}{D_{\mathrm{w,w}}}$		
	median	median	min	max	median	min	max
$V_{5\mathrm{Gy}}$	16	-3.5	-8.6	-1.5	-4.1	-9.4	-2.4
$V_{10\mathrm{Gy}}$	7	-2.3	-5.5	-0.7	-3.1	-6.2	-1.5

Table S2 Comparison of $D_{\mathrm{w,w}}$ and $D_{\mathrm{w,m}}$ as well as $D_{\mathrm{w,w}}$ and $D_{\mathrm{m,m}}$ for $D_{1\mathrm{cc}}$ of the selected OARs. The list of OARs for each case can be found in Table ??. The number of cases containing each OAR is listed in column 2. The $D_{\mathrm{w,w}}$-value is given in Gy, the deviations from $D_{\mathrm{w,w}}$ in percentage of the $D_{\mathrm{w,w}}$-value.

OAR	count	$D_{1\mathrm{cc;w,w}}$ (Gy)	$\frac{D_{1\mathrm{cc;w,m}}-D_{1\mathrm{cc;w,w}}}{D_{1\mathrm{cc;w,m}}}$ (%)			$\frac{D_{1\mathrm{cc;m,m}}-D_{1\mathrm{cc;w,w}}}{D_{1\mathrm{cc;w,m}}}$ (%)		
		median	median	min	max	median	min	max
bile duct	3	12.9	-2.4	-3.0	-1.3	-3.1	-3.6	-2.1
bowel	7	8.0	-2.7	-5.7	-1.3	-3.5	-6.7	-2.1
colon	7	5.7	-3.3	-4.8	-2.1	-4.1	-8.9	-2.9
duodenum	5	7.1	-2.7	-5.3	-1.0	-3.3	-6.1	-2.6
esophagus	4	5.3	-4.2	-7.4	-0.8	-5.1	-8.6	-1.5
gall bladder	1	11.5	-2.7	-	-	-3.7	-	-
heart	8	9.0	-2.6	-12.4	-0.5	-3.4	-13.1	-1.2
kidney	5	17.4	-1.6	-1.7	-1.1	-2.3	-2.6	-1.9
stomach	14	8.0	-2.4	-10.7	-0.4	-2.8	-11.7	-1.2

Table S3 Comparison of $D_{\mathrm{w,w}}$ and $D_{\mathrm{w,m}}$ as well as $D_{\mathrm{w,w}}$ and $D_{\mathrm{m,m}}$ for CTV DVH parameters. The number of treated lesions for each case can be found in Table ??. The total number of treated lesions over all cases was 41. The $D_{\mathrm{w,w}}$-value is given in percentage of the total CTV volume, or percentage of the prescribed dose for volume and dose parameters, respectively. The deviations are shown in percentage of the $D_{\mathrm{w,w}}$-value.

parameter (%)	$D_{\mathrm{w,w}}$	$\frac{D_{\mathrm{w,m}}-D_{\mathrm{w,w}}}{D_{\mathrm{w,w}}}$			$\frac{D_{\mathrm{m,m}}-D_{\mathrm{w,w}}}{D_{\mathrm{w,w}}}$		
	median	median	min	max	median	min	max
V_{150}	85	-0.5	-1.7	0.05	-1.1	-2.3	0
V_{100}	100	-0.05	-0.9	0.01	-0.1	-1.2	0
V_{95}	100	-0.02	-0.7	0.03	-0.06	-1.1	0
V_{90}	100	-0.01	-0.7	0	-0.02	-1.0	0
D_{95}	123	-0.7	-2.7	0.2	-1.5	-3.5	-0.6
D_{90}	139	-0.6	-2.4	0.03	-1.4	-3.1	-0.7
